# Melatonin Mitigates Chilling-Induced Oxidative Stress and Photosynthesis Inhibition in Tomato Plants

**DOI:** 10.3390/antiox9030218

**Published:** 2020-03-06

**Authors:** Meiling Wang, Shuoxin Zhang, Fei Ding

**Affiliations:** 1 School of Life Sciences, Liaocheng University, Liaocheng 252000, China; wangmeiling2081@163.com; 2 College of Forestry, Northwest A&F University, Yangling 712100, China

**Keywords:** melatonin, antioxidant, oxidative stress, chilling stress, photosynthesis, tomato plants

## Abstract

Melatonin has been demonstrated to play a variety of roles in plants. Of particular importance is its role as a potent antioxidative agent. In the present study, we generated melatonin-deficient tomato plants using virus-induced gene silencing (VIGS) approach and melatonin-rich tomato plants by foliar application of melatonin. These tomato plants were used to assess the effect of melatonin on chilling-induced oxidative stress and chilling-induced photosynthesis inhibition. We found that melatonin deficiency increased accumulation of reactive oxygen species (ROS) and aggravated lipid peroxidation in chilling-stressed tomato leaves, while exogenous application of melatonin had the opposite effect. Under chilling stress, melatonin-deficient tomato plants showed impaired antioxidant capacity as evidenced by lower activities of antioxidant enzymes and decreased rations of reduced glutathione (GSH)/oxidized glutathione (GSSG) and reduced ascorbate (AsA)/oxidized ascorbate (DHA), compared with melatonin-rich tomato plants. Furthermore, suppression of melatonin biosynthesis led to more photosynthesis inhibition under the chilling condition and compromised the capability of subsequent photosynthesis recovery in tomato plants. In addition, melatonin-deficient tomato plants displayed less activity of an important Calvin-Benson cycle enzyme sedoheptulose-1,7-bisphosphatase (SBPase) than melatonin-rich tomato plants under chilling stress. Collectively, our data indicate that melatonin is critical for antioxidant capacity and redox balance and is in favor of photosynthesis in tomato plants under chilling stress.

## 1. Introduction

Due to their sessile nature, plants are constantly challenged by a broad spectrum of environmental factors, including low temperature, heat, drought, salinity and heavy metal toxicity. Low temperatures are often defined as low but not freezing temperatures (0–15 °C), which occur frequently in nature during the growth season of warm-climate crops. Low temperatures impair growth and development in crops, causing a considerable proportion of yield loss [1,2]. The primary consequence of low temperature stress is the excessive accumulation of reactive oxygen species (ROS), which consist of hydrogen peroxide (H_2_O_2_), superoxide anion radicals (O_2_**•**^–^), and hydroxyl radicals (•OH), due to disrupted electron transport chain in chloroplasts [3,4]. Excessive accumulation of ROS is toxic to nucleic acids, proteins and lipids, which ultimately causes cellular dysfunction [4,5]. Over-produced ROS also suppresses carbon fixation by inactivating the Calvin–Benson cycle enzymes, resulting in reduced photosynthesis [6,7]. The Calvin–Benson cycle is vital for plant metabolism, generating intermediates required for the biosynthesis of sucrose, starch, isoprenoids and shikimic acid. Thus, low temperature-induced alterations in the enzymes of the Calvin–Benson cycle also impact plant metabolism.

Plants have evolved multiple strategies to cope with low temperature stress. In response to elevated levels of ROS, plants initiate an efficient detoxifying network coordinating antioxidant enzymes and non-enzymatic compounds. Well-known antioxidant enzymes include superoxide dismutase (SOD), catalase (CAT), peroxidase (POD) and ascorbate peroxidase (APX), while representative non-enzymatic compounds comprise glutathione, ascorbic acid, proline, etc. Moreover, a plethora of metabolites, including carbohydrates and amino acids, are generally accumulated to protect plants against chilling injury. These metabolites are important compatible solutes that are required for chilling and drought responses in plants [8,9].

Melatonin (*N*-acetyl-5-methoxytryptamine) is a tryptophan-derived compound, mediating diverse biological processes in humans and animals. Two independent research groups, for the first time, identified the existence of melatonin in higher plants in 1995 [10,11]. Since then, melatonin has received widespread attention among plant biologists and significant advances have been made in understanding of melatonin biosynthetic pathways and its functions in plants. A common biosynthetic pathway of melatonin in many plant species occurs from tryptophan through four sequential enzymatic steps involving tryptophan decarboxylase (TDC), tryptophan 5-hydroxylase (T5H), serotonin *N*-acetyltransferase (SNAT) and acetyl serotonin methyl transferase (ASMT, also known as hydroxy indole-*O*-methyltransferase), among which TDC catalyzes the first step in the pathway [12]. Recent studies have highlighted melatonin as a molecule involved in diverse aspects of developmental and physiological processes, such as seed germination, growth, senescence, photosynthesis, and stress responses [13,14,15,16,17,18,19]. As in animals, the most distinguished role of melatonin in plants is to act as an antioxidant agent in the control of ROS. A growing body of evidence substantiates the idea of melatonin as a common antioxidant, capable of mitigating cellular oxidative stress by directly interacting with ROS and thus stabilizing biological membranes [20,21,22]. In addition to being a direct antioxidant compound, melatonin is able to trigger the expression of genes encoding antioxidant enzymes, such as SOD and CAT, and enhance activities of these enzymes under various stress conditions [13,14,23,24].

Melatonin also acts on photosynthesis in plants under stress conditions by regulating many aspects of photosystems, electron transport chain, and the Calvin–Benson cycle. Melatonin treatment leads to enhanced photosynthetic rate, quantum yield of photosystem II, electron transport rate and non-photochemical quenching in plants under salinity, chilling and drought stresses [25,26]. In the Calvin–Benson cycle, melatonin functions in the regulation of ribulose bisphosphate carboxylase/oxygenase (Rubisco), glyceraldehyde-3-phosphate dehydrogenases, and sedoheptulose-1,7-bisphosphatase (SBPase) [14,21]. We have previously shown that SBPase is severely inhibited by chilling-induced oxidative stress and as a consequence, photosynthetic carbon fixation is decreased [6,27,28]. It is established that melatonin relieves low temperature-induced suppression of photosynthesis in plants originating from tropical/subtropical regions. However, melatonin action on photosynthetic carbon fixation under chilling stress is still not fully understood. In an attempt to uncover the role of melatonin in oxidative stress and photosynthetic carbon fixation in low temperature-stressed tomato plants, we generated melatonin-deficient tomato plants using a virus-induced gene silencing (VIGS) approach. We assessed the effect of melatonin on antioxidant capacity, SBPase activity, and carbon fixation in chilling-stressed tomato plants. Our results suggest that melatonin deficiency aggravates oxidative stress and inhibition of photosynthesis in tomato plants exposed to chilling temperature. Melatonin deficiency also retards the subsequent recovery of photosynthesis when chilling stress is ceased, whereas exogenous application of melatonin has the opposite effect. Our results support the importance of melatonin in maintaining redox homeostasis and photosynthesis in plants under stress.

## 2. Materials and Methods

### 2.1. Plant Materials and Treatment

Tomato (*Solanum lycopersicum* L. cv. Micro-Tom) seeds were germinated at 25 °C in the dark on filter paper in Petri dishes. Germinated seeds were planted in plastic pots (12 cm × 12 cm × 10 cm) containing peat and vermiculite (3/1, v/v). The growth conditions in the growth room were as follows: 400 μmol mol^−1^ CO_2_, 600 μmol m^−2^ s^−1^ photon flux density, 25/20 °C day/night, mean relative humidity 65% and a photoperiod of 14 h.

At the 6-leaf stage, tomato leaves were treated by spraying 100 µM melatonin (MT, Sigma-Aldrich, St. Louis, MO, USA) or mock solution 24 h prior to chilling stress. Melatonin solution was prepared in ethanol and was then diluted in water, with the ethanol/water (v/v) being 1/10,000. The mock solution contained 1/10,000 ethanol. Then, plants sprayed with mock solution were randomly divided into two groups, one of which were grown at 25 °C (control), and the other was placed at 4 °C for 12 h (chilling). Plants pretreated with melatonin were also split into two groups, with one group being grown at 25 °C (MT) and the other being maintained at 4 °C for 12 h (MT + chilling). There were three replicates for each treatment and each replicate consisted of 10 TRV plants and 10 TRV-*TDC* plants. For photosynthesis recovery analysis, plants from the chilling group and the MT + chilling group were placed at 25 °C for 10 h.

### 2.2. Virus-Induced Gene Silencing of TDC

Gene silencing of *tryptophan decarboxylase* (*TDC*) was conducted using the tobacco rattle virus 1 (TRV1) and TRV2 vectors according to the protocol previously described [29]. The 300 bp fragments of *TDC* were amplified using the gene-specific primers. The amplified fragment was digested and ligated into the TRV2 vector. Empty TRV2 vector was used as a control. All constructs were transformed into *Agrobacterium tumefaciens* (GV3101). A mixed culture of *Agrobacterium tumefaciens* harboring a TRV1:TRV2-*TDC* at a 1:1 ratio was infiltrated into the fully expanded cotyledons of tomato plants. The inoculated plants were maintained in a climate chamber for 4 weeks and *TDC* expression was determined using quantitative RT-PCR. Plants with approximately 30% expression level of TRV plants were used for further experiments.

### 2.3. Quantification of Melatonin Content by High-Performance Liquid Chromatography (HPLC)

Melatonin was quantified following a previously reported protocol [30]. Tomato leaves were ground into a fine powder and melatonin was extracted with chloroform at 4 °C in the dark for 12 h. Then the samples were centrifuged at 4000× *g* for 5 min and the chloroform fraction was purified using a C18 solid-phase extraction (SPE) cartridge (Waters, Milford, MA, USA). The extract was evaporated to dryness and dissolved in methanol for analysis using HPLC.

### 2.4. Determination of Transcript Abundance by Quantitative Real-Time PCR

Total RNA was extracted from leaves subjected to different treatments and was used for cDNA synthesis. Quantitative real-time PCR was performed to determine transcript abundance using a Premix Ex Taq kit (TaKaRa, Dalian, China) according to manufacturer’s instructions. Each real-time PCR reaction was performed on iQ5 Multicolor Real-Time PCR Detection System (BIO-RAD, Hercules, CA, USA) and the program is 1 cycle of 30 s at 95 °C, followed by 40 cycles of 5 s at 95 °C, 30 s at 60 °C.

### 2.5. Quantification of H_2_O_2_ and O_2_•^−^

Measurement of H_2_O_2_ was performed according to a previous protocol with modifications [31]. Briefly, leaf samples (0.3 g) were homogenized with 5% (w/v) trichloroacetic acid in an ice bath. The homogenate was centrifuged at 12,000× *g* and the supernatant was mixed with TiCl_2_. The Ti–H_2_O_2_ complex was then precipitated using ammonia solution. The precipitate was collected and resuspended in H_2_SO_4_. The absorbance of the solution was measured at 410 nm and the concentration of H_2_O_2_ was calculated based on a standard curve with different H_2_O_2_ concentrations.

The production of O_2_•^−^ was measured as previously described [32]. Leaf samples were homogenized in phosphate buffer (pH 7.8) and the homogenate was centrifuged at 10,000× *g* for 10 min. The supernatant was then mixed with hydroxylamine hydrochloride. The mixture was incubated at 25 °C for 1 h, and was then mixed with sulfanilamide and α-naphthylamine at 25 °C for 20 min. The absorbance at 530 nm was determined and the production of O_2_•^−^ was calculated.

### 2.6. Assay of Antioxidant Enzyme Activities

Leaf samples were ground with 50 mM potassium phosphate buffer (pH 7.0) containing 0.1 mM EDTA and 1% polyvinylpyrrolidone (w/v). The extract was centrifuged and the supernatant was used for the determination of activities of SOD and CAT as previously described [33,34,35]. SOD activity was assayed by measuring its ability to inhibit the photochemical reduction of nitro blue tetrazolium (NBT). The absorbance was monitored at 560 nm. One unit of SOD is the amount of enzyme required for 50% inhibition of the reduction rate of NBT. CAT activity was measured as the decline in absorbance at 240 nm due to the decline of extinction of H_2_O_2_. The reaction was started by adding H_2_O_2_ to the reaction mixture containing sodium phosphate buffer (pH 7.0), 10 mM H_2_O_2_ and 0.1 ml enzyme extract.

### 2.7. Determination of Malonaldehyde (MDA) Content

Tomato leaves were collected following a 12 h chilling treatment to determine MDA content. Measurement of MDA level was performed as previously described [14,36]. In brief, leaf samples (0.3 g) were homogenized in 3 mL of 0.1% (w/v) trichloroacetic acid. The homogenate was centrifuged at 10,000× *g* for 10 min and 3 mL of 20% TCA containing 0.5% (w/v) 2-thiobarbituric acid was added to 1mL of supernatant. The mixture was heated at 95 °C for 30 min and was then placed in ice bath to stop the reaction. The cooled mixture was centrifuged at 10,000× *g* for 10 min, and the absorbance of the supernatant at 532 nm and 600 nm was recorded.

### 2.8. Determination of Electrolyte Leakage

Tomato leaves were collected at 0 h, 2 h, 4 h, 6 h, 8 h, 10 h and 12 h during chilling treatment in the light to determine the electrolyte leakage. Electrolyte leakage was measured as previously described [37].

### 2.9. Quantification of AsA and GSH

The content of AsA was determined as previously described [38]. AsA was extracted by grinding 0.1 g leaf samples in 6% (v/v) cold HClO_4_. After centrifuging the crude extract, the supernatant was collected for further analysis. AsA content was measured by determining the absorbance difference of the supernatant at 265 nm in 200 mM sodium acetate buffer (pH 5.6) before and after 15-min incubation with 1.5 units of AsA oxidase.

Determination of reduced glutathione (GSH) and oxidized glutathione (GSSG) content was performed according to a previous protocol [39]. Leaf samples were homogenized with 3% trichloroacetic acid containing 0.5 mM EDTA and the homogenates were then centrifuged at 14,000× *g* for 10 min. The supernatant was added to the reaction solution containing 50 mM potassium phosphate, 0.2 mM 5,5′-dithiobis-(2-nitrobenzoic acid) (DTNB), 0.2 mM NADPH, and three units of glutathione reductase. The calculation of total glutathione (GSH + GSSG) was based on the decomposition of DTNB monitored at 412 nm for 1 min. For measurement of GSSG, 2-vinylpyridine was added in the extract and followed the same procedure for total glutathione. The content of GSH was obtained by subtracting GSSG from total glutathione.

### 2.10. Determination of Carbon Assimilation Rates and Quantum Efficiency of Photosystem II

Photosynthetic rates were measured with a portable photosynthesis system (LI-COR 6400, Biosciences, Lincoln, NE, USA). Photosynthetic measurements were performed on new fully expanded leaves. Tomato plants were dark adapted for 30 min and the minimal fluorescence from a dark-adapted leaf (Fo) was measured with a portable fluorometer (PAM-2000, Walz, Germany) and following a saturating pulse, the maximal fluorescence from a dark-adapted leaf (Fm) was obtained, allowing to calculate the maximum quantum efficiency (Fv/Fm).

### 2.11. Assay of SBPase Activity

SBPase activity was determined as previously described [40]. SBPase was extracted from leaf samples in extraction buffer containing 50 mM HEPES, pH 8.2; 5 mM MgCl_2_; 1 mM EDTA; 1 mM EGTA; 10% glycerol; 2 mM benzamidine; 2 mM amino caproic acid; 0.5 mM phenylmethylsulfonyluoride (PMSF); and 10 mM dithiothreitol (DTT). The crude extract was centrifuged, and the supernatant was desalted using a NAP-10 column (GE Healthcare Life Sciences, Pittsburgh, PA, USA) prior to activity assay. For the assay, to start the reaction, 20 μL of protein sample was added to 80 μL of assay buffer (50 mM Tris, pH 8.2; 15 mM MgCl_2_; 1.5 mM EDTA; 10 mM DTT; 2 mM SBP) and incubated at 25 °C for 5 min. The reaction was stopped by the addition of 50 μl of 1 M perchloric acid. The samples were then centrifuged for 5 min and the supernatant assayed for phosphate. Samples (50 µL at 0.5 mM) and phosphate standards (0–0.5 mM NaH_2_PO_4_) were incubated with 850 μL molybdate solution (0.3% ammonium molybdate in 0.55 M H_2_SO_4_) for 10 min at room temperature. Malachite green (0.035% malachite green, 0.35% polyvinyl alcohol) was added (150 μL) and the samples were incubated for a further 45 min at room temperature before measuring the absorbance at 620 nm with UV-visible spectrophotometer (UV-2450, Shimadzu Corporation, Kyoto, Japan).

### 2.12. Statistical Analysis

All experiments in this study were repeated three times, and the values presented were the means ± standard deviations (SDs). Duncan’s multiple range test was performed to compare the difference among treatments. Different letters in figures indicate significant difference at *p* < 0.05.

## 3. Results

### 3.1. Low Temperatures and Exogenous Melatonin Induce the Expression of Melatonin Biosynthetic Gene TDC

**Figure 1:**
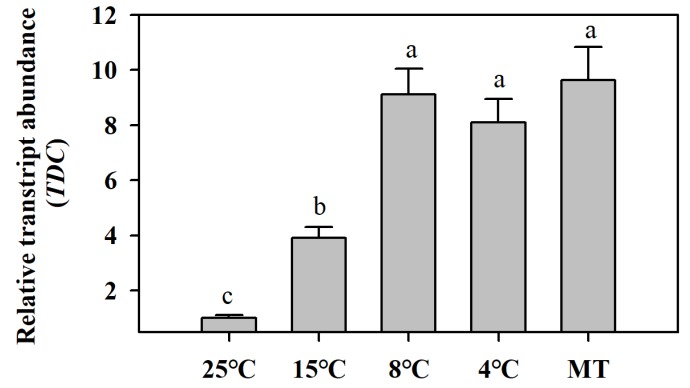
Low temperatures and exogenous melatonin induce the expression of melatonin biosynthetic gene *TDC*. Tomato plants at the 6-leaf stage were exposed to different low temperatures for 12 h and leaf samples were collected for the determination of chilling-induced *TDC* expression by quantitative RT-PCR. Exogenous melatonin (MT, 100 μM) was sprayed on tomato leaves 12 h prior to the collection of leaves for analysis of *TDC* transcript abundance by quantitative RT-PCR. The expression level of *TDC* in tomato leaves at 25 °C was set to 1, and the relative expression levels in the rest of samples were calculated accordingly. The values presented are means ± standard deviations (SDs; *n* = 3). Different letters (a,b,c) above bars indicate significant difference at *p* < 0.05 among treatments.

To study how melatonin biosynthesis was alerted in response to low temperatures and exogenous melatonin, we first investigated the expression level of *TDC* gene, which encodes the enzyme catalyzing the first step of melatonin biosynthesis in plants. The results showed that expression of *TDC* was induced by low temperatures, with the strongest induction being at 8 °C. Expression of *TDC* was also markedly induced by application of exogenous melatonin (Figure 1). These results indicate that the *TDC* gene may play a critical role in melatonin biosynthesis in response to chilling temperatures and exogenous melatonin.

### 3.2. Melatonin Accumulation is Altered in TDC-Silenced and Melatonin-Treated Tomato Leaves

**Figure 2:**
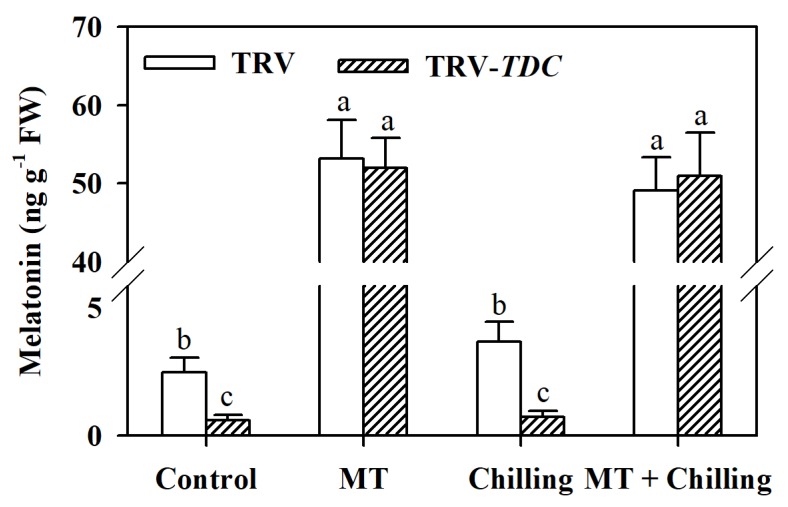
Silencing *TDC* suppresses melatonin biosynthesis, while exogenous melatonin increases melatonin accumulation in tomato leaves. Tomato plants at the 6-leaf stage were exposed to chilling stress (4 °C) for 12 h and leaf samples were collected for the determination of melatonin content by HPLC. FW stands for fresh weight. TRV were those plants without *TDC* silencing. The values presented are means ± SDs (*n* = 3). Different letters (a,b,c) above bars indicate significant difference at *p* < 0.05 among treatments.

To investigate the importance of melatonin in the reduction of chilling-induced oxidative stress in tomato plants, we silenced the melatonin biosynthetic gene *TDC* using VIGS. Compared with TRV plants, TRV-*TDC* plants showed a reduced level of melatonin under both control and chilling conditions, demonstrating that *TDC* was efficiently suppressed in tomato plants. However, inconsistent with the result that chilling dramatically induced expression of *TDC* gene, only slightly higher melatonin accumulation was observed in TRV plants following 12-h chilling stress (Figure 2). To further reveal the role of melatonin in the oxidative stress tolerance, we applied melatonin to tomato leaves. The results showed that foliar application of melatonin dramatically enhanced the accumulation of melatonin in leaves of both TRV plants and TRV-*TDC* plants under chilling and control conditions (Figure 2). Thus, we have generated melatonin-deficient tomato plants by silencing the *TDC* gene and melatonin-rich plants by exogenous application of melatonin and these plants were used to assess the effects of melatonin on chilling-induced oxidative stress.

### 3.3. Melatonin Decreases Chilling-Induced ROS Accumulation in Tomato Leaves

**Figure 3:**
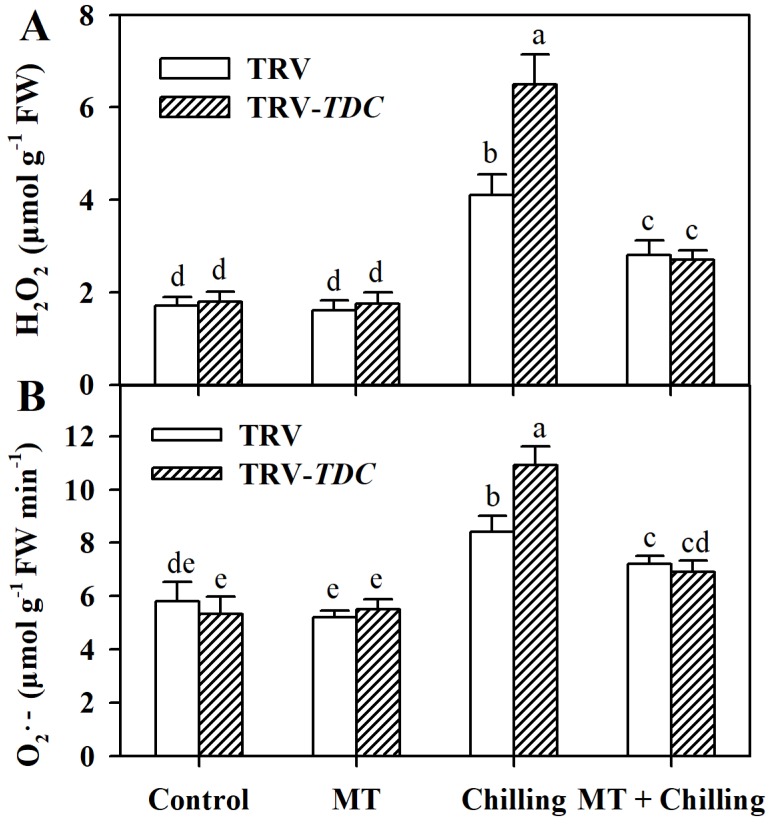
Accumulation of hydrogen peroxide and superoxide anion as affected by *TDC* silencing and exogenous melatonin in tomato plants under chilling stress. (**A**) H_2_O_2_ level; (**B**) O_2_**•**^–^ level. Tomato plants at the 6-leaf stage were exposed to chilling stress (4 °C) for 12 h and leaf samples were collected for the determination of reactive oxygen species (ROS) levels. The values presented are means ± SDs (*n* = 3). Different letters (a–e) above bars indicate significant difference at *p* < 0.05 among treatments.

To investigate the alleviating effect of melatonin on low temperature-induced oxidative stress, we quantified H_2_O_2_ content in TRV plants, TRV-*TDC* plants and melatonin-treated plants under chilling stress. It was observed that chilling stress led to a dramatic increase in the level of H_2_O_2_ in TRV plants, while the application of exogenous melatonin reduced the accumulation of H_2_O_2_. Notably, the accumulation of H_2_O_2_ in *TDC*-silenced plants was significantly higher than that in TRV plants and melatonin-treated plants under chilling conditions (Figure 3A). Similarly, production of O_2_**•**^–^ in TRV-*TDC* plants was also increased in comparison with that in TRV plants under chilling stress (Figure 3B). These results indicate that melatonin favors the removal of chilling-induced excessive production of ROS in tomato leaves.

### 3.4. Melatonin Alleviates Chilling-Triggered Oxidative Damage to Cell Membranes in Tomato Leaves

**Figure 4:**
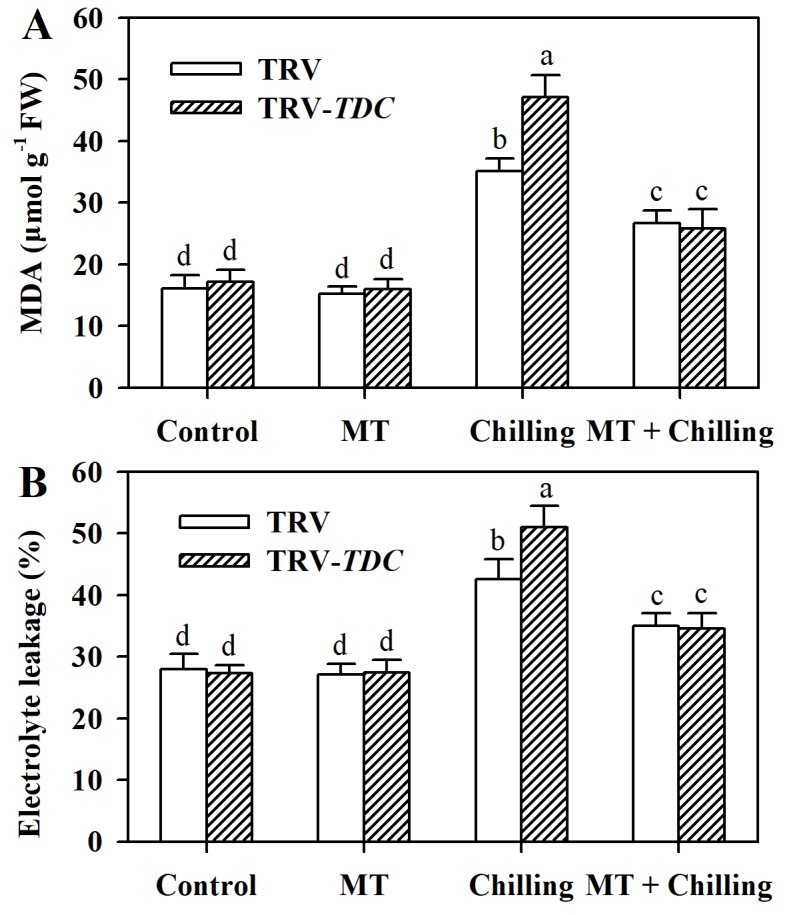
Lipid peroxidation and membrane stability as affected by *TDC* silencing and exogenous melatonin in tomato leaves under chilling stress. (**A**) MDA content; (**B**) electrolyte leakage. Tomato plants at the 6-leaf stage were exposed to chilling stress (4 °C) for 12 h and leaf samples were collected for the determination of MDA and electrolyte leakage. The values presented are means ± SDs (*n* = 3). Different letters (a–d) above bars indicate significant difference at *p* < 0.05 among treatments.

One of the harmful effects of low temperature stress is the oxidative damage to cell membranes. To examine the role of melatonin in membrane stability, we next measured malonaldehyde (MDA) content and electrolyte leakage in tomato leaves subjected to chilling stress. MDA content and electrolyte leakage were markedly increased in chilling-stressed plants compared with plants grown at optimal temperature. It was of note that under chilling stress, silencing *TDC* aggravated lipid peroxidation by enhancing MDA content and electrolyte leakage, whereas pretreatment of tomato plants with melatonin significantly relieved lipid peroxidation (Figure 4).

### 3.5. Melatonin Enhances Antioxidant Capacity in Tomato Leaves under Chilling Stress

**Figure 5:**
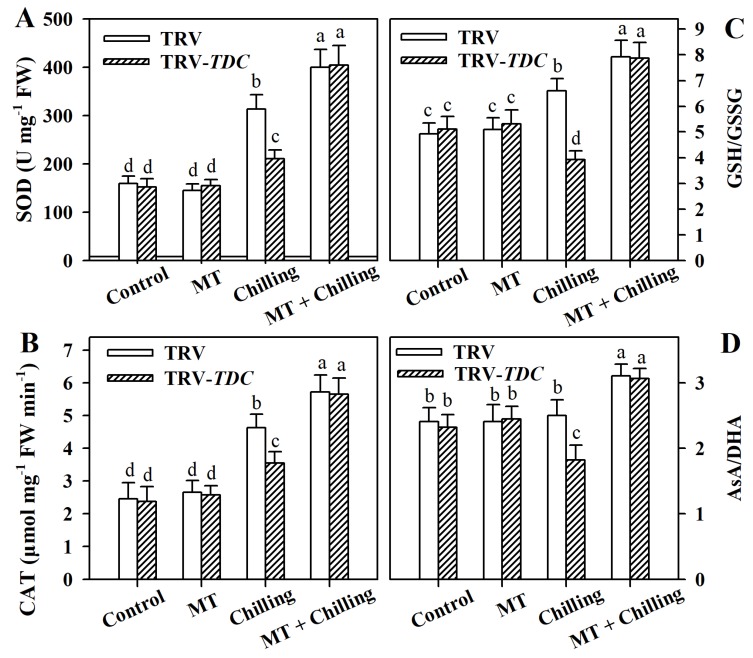
Changes in the antioxidant enzymes and non-enzymatic compounds as affected by *TDC* silencing and exogenous melatonin in tomato plants under chilling stress. (**A**) Superoxide dismutase (SOD); (**B**) catalase (CAT); (**C**) ratio of GSH/GSSG; (**D**) ratio of AsA and DHA. Tomato plants at the 6-leaf stage were exposed to chilling stress (4 °C) for 12 h and leaf samples were collected for the determination of antioxidant enzyme activities and non-enzymatic compound levels. The values presented are means ± SDs (*n* = 3). Different letters (a–d) above bars indicate significant difference at *p* < 0.05 among treatments.

Plants have developed a sophisticated antioxidant system, consisting of antioxidant enzymes and non-enzymatic compounds, to cope with oxidative stress. To investigate the impact of melatonin on the antioxidant system, we assayed activities of typical antioxidant enzymes SOD and CAT, and calculated the rations of GSH/GSSH and AsA/DHA. The results showed that under chilling stress, both SOD and CAT activities were enhanced in TRV plants, and addition of exogenous melatonin further stimulated these activities. However, compared with TRV plants, TRV-*TDC* plants showed reduced activities of SOD and CAT under chilling stress (Figure 5A,B).

Glutathione and ascorbate are important for redox homeostasis in plants. We thus performed an analysis of oxidized and reduced forms of glutathione and ascorbate and calculated their respective ratios. Under chilling stress, GSH/GSSG ratio was increased in TRV plants, whereas the ratio was decreased in *TDC*-silenced plants. Likewise, AsA/DHA ratio was decreased in TRV-*TDC* plants compared with that in TRV plants under chilling stress (Figure 5C,D). However, AsA/DHA in TRV plants under chilling conditions was not significantly different from that in TRV plants under the control condition. These results demonstrate that melatonin exerts a positive influence on the antioxidant capacity of chilling-stressed tomato leaves.

### 3.6. Melatonin Increases Photosynthetic Carbon Assimilation and Activity of Calvin–Benson Cycle Enzyme SBPase in Chilling-Stressed Tomato Plants

**Figure 6:**
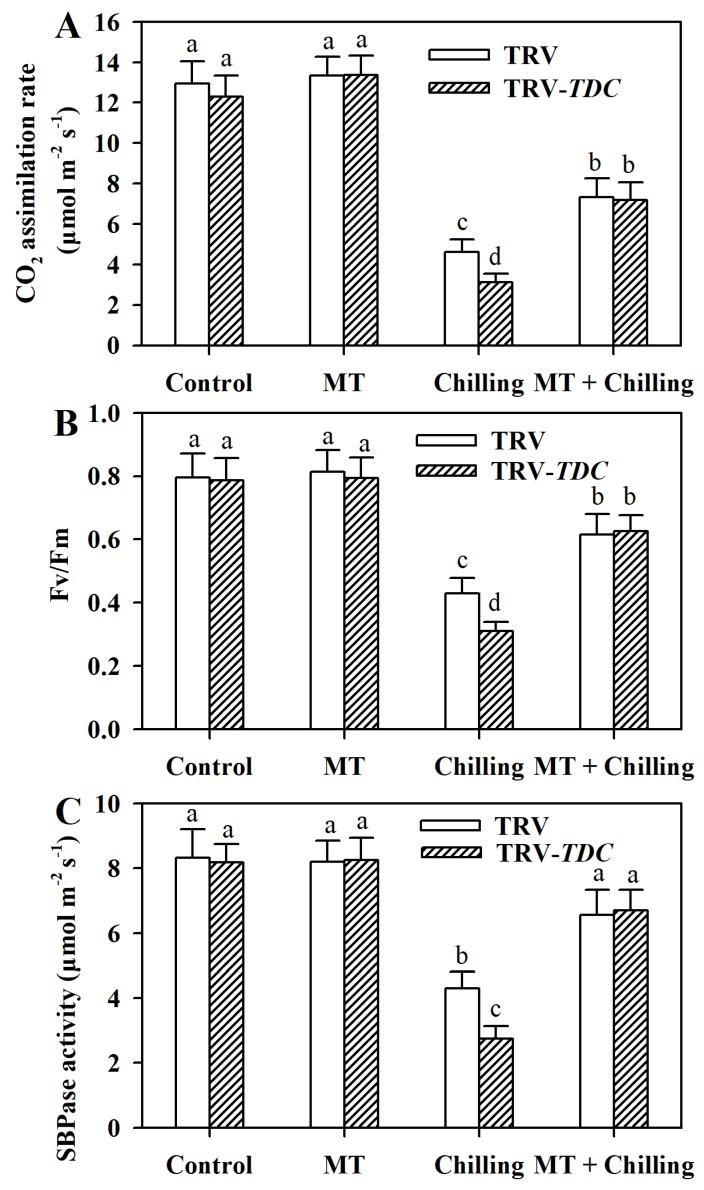
Changes in photosynthesis. (**A**) Carbon assimilation rate; (**B**) Maximum quantum yield of photosystem II (PSII; Fv/Fm); (**C**) activity of Calvin–Benson cycle enzyme SBPase as affected by *TDC* silencing and exogenous melatonin in tomato leaves under chilling stress. Tomato plants at the 6-leaf stage were exposed to chilling stress (4 °C) for 12 h and leaf photosynthesis was measured using a portable photosynthesis system. Leaf samples were collected for the determination of SBPase activity. The values presented are means ± SDs (*n* = 3). Different letters (a–d) above bars indicate significant difference at *p* < 0.05 among treatments.

Photosynthesis has been shown to be highly sensitive to low temperatures [3]. Melatonin is known to promote photosynthesis under various stress conditions. To unveil the effect of melatonin on photosynthetic carbon fixation in chilling-stressed tomato plants, we analyzed the carbon assimilation rates and maximum quantum yield of photosystem II (PSII; Fv/Fm). The results showed that carbon assimilation rates and Fv/Fm were inhibited by chilling stress; however, significant decreases in carbon assimilation rates and Fv/Fm were observed in *TDC*-silenced plants compared with those in TRV plants under chilling stress. Contrary to *TDC* silencing, exogenous application of melatonin boosted carbon assimilation and Fv/Fm in tomato plants exposed to chilling stress (Figure 6A,B).

SBPase is an enzyme involved in carbon fixation [41]. Previous studies have shown that this enzyme is susceptible to chilling temperatures [6,42]. Our results showed that following a 12-h chilling treatment, SBPase activity was severely inhibited in TRV plants and *TDC*-silenced plants, while melatonin pretreatment relieves the inhibition (Figure 6C). Importantly, *TDC*-silenced plants displayed lower SBPase activity than TRV plants under chilling conditions. The results imply that melatonin may be accountable for the increased carbon flux by increasing SBPase activity under chilling stress.

### 3.7. Melatonin Facilitates the Recovery of Photosynthesis in Chilling-Stressed Tomato Plants

**Figure 7:**
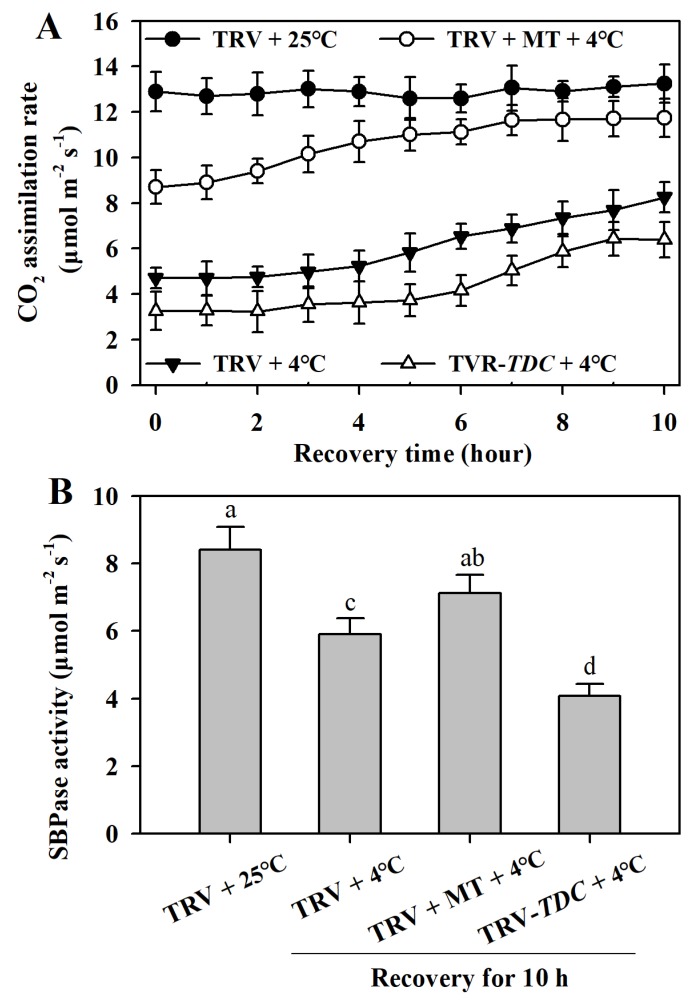
Melatonin accelerates the recovery of photosynthesis (**A**) and the restoration of SBPase activity (**B**) in chilling-stressed tomato plants. Following a 12-h chilling stress, tomato plants were placed back at 25 °C for 10 h for the determination of photosynthesis recovery. Photosynthetic carbon assimilation rates of tomato leaves were measured at 1 h intervals. SBPase activities were assayed at hour 10. The values presented are means ± SDs (*n* = 3). Different letters (a–d) above bars indicate significant difference at *p* < 0.05 among treatments.

Once stress ceases, the fast recovery of photosynthesis is critical for the survival of plants. We thus measured the carbon assimilation rates of tomato plants, which were subsequently moved to normal growth temperature after 12 h chilling stress, to examine effect of melatonin on the capacity for photosynthesis recovery. The results showed that following chilling stress, photosynthesis of all tomato plants could not be fully restored; however, photosynthesis in melatonin-treated plants showed a quick recovery and could be restored to 90% of that in plants without chilling stress. In contrast, photosynthesis in *TDC*-silenced plants manifested a slow recovery and could only be restored to 66% of that in plants without chilling stress (Figure 7A). These results suggest that melatonin aids photosynthesis recovery after chilling stress.

To further reveal the involvement of melatonin in the recovery of photosynthesis, we assayed SBPase activity after chilling-stressed plants were returned to normal growth temperature. Following exposure to 25 °C for 10 h, melatonin-treated plants with prior chilling stress regained 85% of SBPase activity, while *TDC*-silenced plants restored just 50% of SBPase activity (Figure 7B), suggesting melatonin is vital in the protection of SBPase against low temperature stress.

## 4. Discussion

Low temperature is a common adverse environmental factor during the growth season of many warm-climate crop species. It hampers plant growth and development, and thus causes loss of plant productivity. One of the detrimental consequences of low temperature is the excessive production of ROS, which disrupts redox balance and leads to oxidative stress in plants. Oxidative stress usually causes damage to cell membranes, nucleic acids, proteins and lipids, and impairs many physiological processes. It is therefore critical to keep the production of ROS under control in plants. Melatonin, first identified as an animal hormone, has been demonstrated to play important roles in the protection of plants against a diversity of abiotic stresses, including high/low temperatures, drought, salinity and heavy metal toxicity [25,26,43,44,45,46,47,48]. The most recognized function of melatonin in these cases is to scavenge excessive ROS, thereby maintaining redox homeostasis in plants under stress conditions. The effect of melatonin on cold tolerance has been massively investigated in plants [14,19,24,46], however, most studies rely principally on the exogenous application of melatonin. Additionally, few studies investigate the impact of melatonin on Calvin–Benson cycle enzymes. In the present work, we generated melatonin-deficient tomato plants using the VIGS approach. Together with melatonin-rich plants (exogenous application of melatonin), melatonin-deficient tomato plants were used to investigate the ameliorative effect of melatonin on chilling-induced oxidative stress, photosynthesis inhibition and SBPase inactivation. We have concluded that melatonin was effective in the mitigation of oxidative stress by promoting antioxidant capacity, and the alleviation of chilling-induced photosynthesis inhibition by maintaining PSII activity and enhancing activity of the Calvin–Benson cycle enzyme SBPase. The evidence that we presented includes: (1) reduced level of endogenous melatonin increased the accumulation of ROS and aggravated lipid peroxidation in chilling-stressed tomato leaves, while exogenous application of melatonin had the opposite effect; (2) under chilling stress, melatonin-deficient tomato plants showed less antioxidant capacity as evidenced by lower activities of antioxidant enzymes and decreased ratios of GSH/GSSG and AsA/DHA, compared with melatonin-rich tomato plants; (3) suppression of melatonin biosynthesis led to greater photosynthesis inhibition under chilling conditions and compromised the capability of photosynthesis recovery after chilling stress; (4) melatonin deficiency increased SBPase inactivation and retarded photosynthesis recovery; conversely, melatonin enrichment sustained higher SBPase activity and facilitated photosynthesis recovery in chilling-stressed tomato plants.

In this work, we first performed an expression analysis of the melatonin biosynthetic gene *TDC* in response to low temperature. It was observed that the *TDC* gene was strongly induced by low temperatures. Thus, we expected to see a marked increase in melatonin content in chilling-treated tomato plants. However, surprisingly, following 12 h of chilling treatment, melatonin content was only slightly increased in TRV plants. In an attempt to explain this unexpected result, we speculate that 12 h of chilling period might not be long enough to observe a significant increase of melatonin accumulation, because protein synthesis usually lags behind gene expression. Though *TDC* was dramatically induced after 12 h of chilling treatment, significant accumulation of melatonin may occur hours later. However, the result does not invalidate the use of TRV-*TDC* plants to assess the effect of melatonin on chilling-induced oxidative stress and photosynthesis inhibition, because *TDC* silencing using VIGS led to a significant reduction in melatonin in tomato plants, allowing us to investigate the antioxidative role of melatonin under chilling stress.

The role melatonin as an antioxidant has been well established in plants under a diversity of stress conditions [19]. We found that tomato plants exposed to chilling temperature accumulated more ROS than those grown under normal temperature; however, melatonin pretreatment significantly reduced the accumulation ROS. On the contrary, suppression of melatonin biosynthesis via VIGS increased the production of ROS. These results further support the recognized role of melatonin as an antioxidant. Manipulation of melatonin levels in tomato plants also makes it possible to assess the effect of melatonin on chilling-induced oxidative damage to tomato plants. We observed that exogenous application of melatonin dramatically alleviated lipid peroxidation as indicated by decreased MDA content and electrolyte leakage in chilling-stressed tomato plants, while silencing the melatonin biosynthetic gene *TDC* exacerbated the lipid peroxidation. These results, together with previous findings [13,14,49], suggest the crucial role of melatonin in the protection of membrane against oxidative stress.

ROS homeostasis in plants is largely dependent on an antioxidant network that involves antioxidant enzymes and non-enzymatic compounds. Melatonin has been shown in numerous studies to promote the antioxidant capacity by enhancing the activities of antioxidant enzymes and contents of antioxidant compounds in plants subjected to various stresses [50,51]. Consistently, our work showed that melatonin application significantly elevated the activities of representative antioxidant enzymes SOD and CAT in chilling-stressed tomato leaves; in comparison, reduced melatonin accumulation led to decreased activities of these two enzymes. In this study, we did not examine other antioxidant enzymes such as APX and POD; however, based on our previous work [26] that melatonin enhanced the activities of SOD, CAT, APX and POD in chilling-stressed tomato plants, we speculate that melatonin may also exert a positive influence on the activities of APX and POD in our current work. In addition, the ratios of GSH/GSSH and AsA/DHA were increased in melatonin-treated plants, whereas they were decreased in melatonin-deficient plants under chilling conditions. Thus, these results substantiate that melatonin plays a key role in the regulation of antioxidant capacity in tomato plants exposed to chilling temperatures.

Photosynthesis is a complex process consisting of many biological pathways. Environmental stresses, such as low temperature, drought and salinity, disrupt the processes of photosynthetic carbon metabolism in plants, ultimately restricting growth and yield. Previous studies demonstrate that photosynthesis is highly sensitive to chilling temperatures, with both light reaction and dark reaction being likely to be inhibited [26,42]. Similar results were observed in our study. We found that carbon assimilation rates and Fv/Fm were markedly reduced by chilling stress. However, the inhibitions were alleviated by exogenous application of melatonin. In contrast, they were aggravated by silencing *TDC* under chilling conditions. These results were in line with previous studies [13,14] and support that melatonin protects photosystem II and photosynthesis against low temperature stress.

Resumption of photosynthesis and growth is critical for the survival of stressed plants. In the present study, it was found that melatonin influenced the recovery of photosynthesis after chilling-stressed tomato plants were returned to normal growth temperature. Exogenous melatonin facilitated the recovery, while melatonin deficiency retarded the recovery and prevented the full restoration of photosynthesis. Inactivation of Calvin–Benson cycle enzymes was found to closely mirror the chilling-induced inhibition of photosynthesis in plants [52]. SBPase is a key enzyme in the Calvin–Benson cycle and is required for carbon fixation. It has been well recognized that a small decrease in SBPase activity significantly reduces photosynthesis [7,28,53]. In this study, analysis of SBPase activity revealed that the maximum catalytic activity of SBPase in melatonin-deficient plants after chilling stress was more suppressed compared with that in melatonin-rich plants. The difference in SBPase activity may partly explain the recovery discrepancy of photosynthesis. Different from other Calvin–Benson cycle enzymes, SBPase is sensitive to low temperatures and is vulnerable to oxidative stress [42,54]. Thus, it is likely that melatonin sustains SBPase activity by reducing oxidative stress and facilitates photosynthesis recovery.

## 5. Conclusions

**Figure 8:**
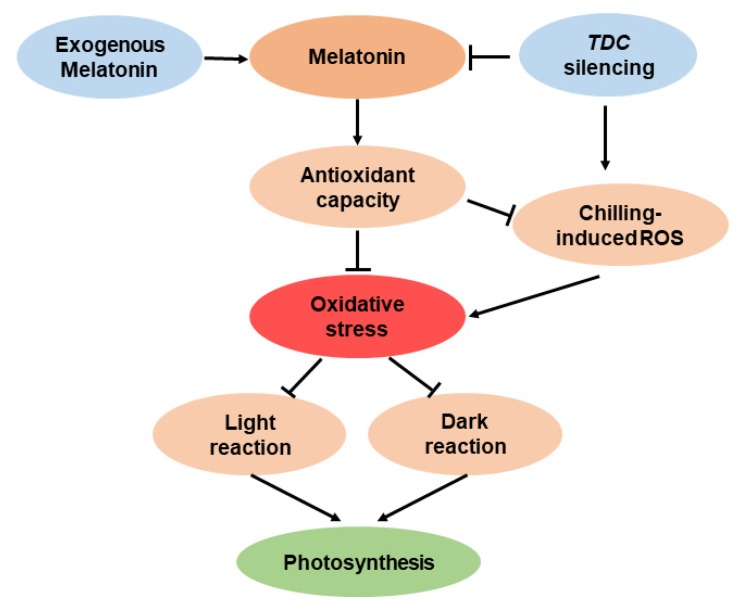
Schematic representation of melatonin in the mitigation of chilling-induced oxidative stress and chilling-induced photosynthesis inhibition in tomato plants. Exogenous melatonin enhances the accumulation of melatonin, which promotes the antioxidant capacity of tomato leaves, thus reducing the chilling-induced oxidative stress. Melatonin-mediated reduction in oxidative stress is advantageous to photosystem II (light reaction) and the Calvin–Benson cycle (dark reaction), thereby favoring photosynthesis under chilling stress. In contrast, silencing *TDC* suppresses melatonin biosynthesis and thus aggravates chilling-induced oxidative stress, leading to inhibited photosynthesis in tomato plants.

In conclusion, using melatonin-rich and melatonin-deficient tomato plants, we have demonstrated that melatonin mitigates chilling-induced oxidative stress, promotes photosynthetic carbon fixation, and facilitates photosynthesis recovery after chilling stress (Figure 8). Melatonin strengthens the antioxidant potentials of chilling-stressed tomato leaves by enhancing antioxidant enzyme activities and the redox pool, reducing ROS accumulation and stabilizing membranes. In addition, melatonin is advantageous to photosynthesis under chilling stress by acting on photosystem II and Calvin–Benson cycle enzymes.
